# The Ubiquitous Dermokine Delta Activates Rab5 Function in the Early Endocytic Pathway

**DOI:** 10.1371/journal.pone.0017816

**Published:** 2011-03-10

**Authors:** Emilie A. Leclerc, Leila Gazeilles, Guy Serre, Marina Guerrin, Nathalie Jonca

**Affiliations:** UMR 5165 “Epidermis Differentiation and Rheumatoid Autoimmunity Unit”, CNRS – University Toulouse III (IFR 150, INSERM – CNRS – University Toulouse III – CHU), CHU Purpan, Toulouse, France; Cornell University, United States of America

## Abstract

The expression of the recently identified dermokine (*Dmkn*) gene leads to four families of proteins with as yet unknown functions. The secreted α, β and γ isoforms share an epidermis-restricted expression pattern, whereas the δ isoform is intracellular and ubiquitous. To get an insight into Dmknδ function, we performed yeast two-hybrid screening and identified the small GTPases Rab5 as partners for Dmknδ. The Rab5 proteins are known to regulate membrane docking and fusion in the early endocytic pathway. GST pull-down assays confirmed the direct interaction between Rab5 and Dmknδ. Transient expression of Dmknδ in HeLa cells led to the formation of punctate structures colocalized with endogenous Rab5 and clathrin, indicating Dmknδ involvement in the early steps of endocytosis. Dmknδ indeed colocalized with transferrin at early stages of endocytosis, but did not modulate its endocytosis or recycling kinetics. We also showed that Dmknδ was able to bind both inactive (GDP-bound) and active (GTP-bound) forms of Rab5 in vitro but preferentially targeted GDP-bound form in HeLa cells. Interestingly, Dmknδ expression rescued the Rab5S34N-mediated inhibition of endosome fusion. Moreover, Dmknδ caused the enlargement of vesicles positive for Rab5 by promoting GTP loading onto the small GTPase. Together our data reveal that Dmknδ activates Rab5 function and thus is involved in the early endosomal trafficking.

## Introduction

The recent identification of the Dermokine (*Dmkn*) gene came from different studies carried out to identify new genes specifically expressed during the late stage of epidermis differentiation [1], [2], [3], [4], [5]. Mapped to human chromosome 19q13.1, *Dmkn* spans 25 exons. Its expression leads to four groups of transcripts according to three different transcriptional start sites, two transcriptional termination sites, and several alternative coding exons [3]. The corresponding isoforms were named α, β, γ and δ. The δ transcripts, spanning from exon 6 to exon 25, are radically different from the α, β and γ transcripts [3]. First, they show a very broad pattern of expression, including numerous tissues and organs [3], [6], [7], [8], whereas α, β and γ mRNAs expression is mainly restricted to epidermis. Second, unlike α-, β- and γ-groups, δ mRNAs do not encode a putative signal peptide and are predicted to produce cytosolic proteins. This was confirmed by the expression of recombinant Dmknδ in transfected 293/EBNA cells [3]. Finally, the δ family of transcripts is represented by a surprisingly broad number of members. We cloned up to 9 different cDNAs from human epidermis, potentially encoding 6 different Dmknδ proteins [3].

Rab proteins make up the largest subfamily of small GTPases that play central roles in intracellular membrane trafficking. So far, in humans, the Rab family has been shown to have more than 60 proteins scattered around distinct intracellular compartments, where they regulate vesicle budding, transport and fusion [9], [10]. Rab proteins cycle between an active (GTP-bound) and an inactive (GDP-bound) state. The nucleotide switch leads to a Rab conformational change which determines the interaction with specific regulators and effectors that are located both on membranes and in the cytosol [11]. For example, the GDP/GTP exchange factors (GEFs) catalyze the conversion from the GDP- to GTP-bound state, whereas GTPase-activating proteins (GAPs) catalyze GTP hydrolysis [12]. Among the Rab family of proteins, Rab5 is a key player in the early endocytic pathway. It regulates clathrin-coated vesicle-mediated transport from the plasma membrane to the early endosomes as well as homotypic early endosome fusion. Moreover, it has also been implicated in endosome motility along microtubules [13] and actin filaments [14] and also in growth factor signalling [15]. The three Rab5 paralogues Rab5a, b and c [16], encode isoforms showing distinct tissue distributions [17]. At least 20 cytosolic proteins specifically interact with active Rab5, highlighting the complexity of the downstream regulation by this GTPase [18].

The Dmknδ share no sequence similarity with any known protein. In order to elucidate its role we thus performed yeast two-hybrid screening and identified the Rab5 proteins as partners. By GST pull-down experiments and confocal microscopy analysis of transiently transfected HeLa cells, we further characterized the involvement of Dmknδ in the early endosomal trafficking.

## Materials and Methods

### Yeast two-hybrid screening

The yeast reporter strain AH109 was sequentially transformed with pGBKT7-Dmknδ5 and a cDNA library (Matchmaker human keratinocyte library in pGAD10, Clontech) following the instructions of the Matchmaker Gal4 two-hybrid system (Clontech). The double transformants were plated on selective medium lacking tryptophan, leucine and histidine and grown at 30°C for 5 days. Positive colonies were then picked, plated on selective medium lacking tryptophan, leucine, histidine and adenine, and tested for β-galactosidase activity using a replica plate assay. About 2.5 million library clones were screened. Library plasmids from positive colonies were isolated using Fast Prep (Thermo Scientific), rescued into E. coli DH5α and sequenced.

### Antibodies

Primary antibodies were: polyclonal anti-Rab5b and anti-Rab7 (Santa Cruz Biotechnology), monoclonal anti-Rab11, anti-LAMP1 and anti-EEA1 (BD Biosciences), monoclonal anti-clathrin (Abcam), monoclonal anti-GST (Cell Signaling Technology) and anti-GFP (Novus Biologicals). Alexa-Fluor- 555 secondary antibodies were obtained from Invitrogen.

### Cell culture and transfection

HeLa cells were cultured in Dulbecco's Modified Eagle's Medium (DMEM) plus GlutaMAX^TM^ supplemented with 10% heat-inactivated foetal bovine serum, 50 U/ml penicillin and 50 µg/ml streptomycin (Invitrogen) at 37°C in 5% CO_2_. HeLa cells were transfected with plasmid constructs using JetPEI reagent (Polyplus Transfection), according to the manufacturer's instructions.

### Plasmid constructs

All cDNA clones used in this study were obtained by polymerase chain reaction (PCR) with specific primers. The DNA sequence of the insert as well as the flanking regions in each cDNA clone was verified by sequencing.

#### Yeast two-hybrid constructs

Dmknδ5 cDNA was generated by PCR with the previously made pCEP4 construct as template [3], and cloned into the pCR2.1TOPO vector (Invitrogen). cDNAs of Dmknδ5, Dmknδ5-Nt (corresponding to exons 13 to 19 of Dmknδ5), and Dmknδ5-Ct (corresponding to exons 20 to 23 of Dmknδ5), were subcloned into the pGBKT7 vector (Clontech). Rab5a cDNA was PCR amplified using the pCMV-SPORT6-Rab5a (purchased from RZPD) as template and cloned into the pGADT7 plasmid (Clontech).

#### Constructs for *in vitro* binding assays

Dmknδ5, Dmknδ5-Nt and Dmknδ5-Ct cDNAs were cloned into the pGEX-6P-1 expression vector (Amersham Biosciences). Wild-type Rab5b (wt) cDNA was amplified by PCR from the pGAD10 construct rescued from yeast two-hybrid screening, and cloned into pCR2.1TOPO. The previously described mutants Rab5S34N and Rab5Q79L [19] were generated by site-directed PCR mutagenesis using Rab5bwt cDNA as template and specific primers harbouring the mutation concerned. Each Rab5b form was subcloned into the pGEX-6P-1 plasmid. The previously described “Rab5 binding domain” (R5BD) comprising the last 73 amino acids of rabaptin-5 [20] was obtained by RT-PCR from total HeLa cells mRNA and cloned into the pGEX-6P-1 plasmid.

#### Constructs for the localization studies

Dmknδ5 and Rab5b constructs were cloned into the pEGFP-C1 and pDsRed1-C1 vectors (Clontech) respectively.

### Recombinant proteins

The pGEX-6P-1 vectors were transformed into E. coli BL21-CodonPlus competent cells (Stratagene) and protein expression was induced with 1 mM isopropyl thio-β-D-galactoside (IPTG) for 2 hours at 37°C. Recombinant GST proteins were then extracted from bacteria cells and purified on a Glutathione Sepharose 4 Fast Flow column (Amersham Biosciences) according to the manufacturer's instructions. GST-Rab5bwt recombinant protein was further treated with PreScission Protease (GE Healthcare) to remove the GST moiety following the manufacturer's recommendations.

### Immunoblotting

Proteins separated by SDS-PAGE were transferred to a Hybond-C extra membrane (GE Healthcare) and probed overnight at 4°C with primary antibodies. Bound antibodies were detected with horseradish peroxidase-conjugated secondary antibodies and developed using the Lumi-Light kit (Roche Applied Science). To determine relative protein amounts, three representative exposures for each sample were quantitated by densitometry analysis using the ImageJ free software.

### HeLa cell protein extract

HeLa cells were harvested 36 hours after transfection in lysis buffer (25 mM Hepes-NaOH pH 7.4, 100 mM NaCl, 5 mM MgCl_2_, 1% NP40, 10% glycerol, 1 mM DTT, protease inhibitors). Extracts were incubated for 5 minutes on ice and clarified by centrifugation (10,000×*g*, 1 minute, 4°C). The supernatants were recovered and used for further pull-down assays.

### GST pull-down assays

20 µg of glutathione sepharose (GS) beads were coated with 30 µg of GST-Dmknδ5 or GST alone for 1 hour at 4°C. After washing and equilibration, Hela cell protein extract or 10 µg of Rab5b recombinant protein were incubated for 1 hour at 4°C with coated beads. Interacting complexes were eluted with 10 mM Gluthation pH 8 and subjected to immunoblotting. In some cases, cleaved Rab5bwt recombinant protein was preloaded with 500 µM of GppNHp, a non-hydrolysable analogue of GTP or 500 µM of GDP (Jena Biosciences), overnight at 4°C, in the presence of 10 mM EDTA and 0.3% β-mercaptoethanol. The nucleotide binding reaction was stopped by adding 10 mM MgCl_2_. GppNHp-bound Rab5bwt and GDP-bound Rab5bwt were used for further pull-down assays.

### GTP-loaded Rab5 pull-down assay

The GST-R5BD pull-down assay was performed as previously described [21]. Briefly, 80 µg of GS beads were coated with 100 µg of GST-R5BD. Beads were then incubated with fresh transfected HeLa cell protein extract for 1 hour at 4°C. Eluted interacting complexes were subjected to immunoblotting.

### Immunofluorescence

Hela cells were grown on glass coverslips for 24 hours and subjected or not to transient transfection. After 36 hours, the cells were fixed with methanol at −20°C for 2 minutes. For indirect immunofluorescence experiments, cells were immunostained with primary antibodies for 1 hour at 37°C. The respective AlexaFluor conjugated secondary antibodies were then incubated for 1 hour at room temperature. After extensive washing with PBS, the coverslips were mounted in Mowiol (Sigma-Aldrich) on glass slides and imaged on a Carl Zeiss confocal microscope LSM710. Final images were analysed using the Zen software (Carl Zeiss).

### Transferrin internalization assay

To deplete endogenous transferrin, HeLa cells transiently expressing GFP-Dmknδ5 or not were serum-starved for 2 hours at 37°C in internalization medium (IM) consisting of DMEM with 20 mM Hepes-NaOH (pH 7.4) and 2 mg/ml BSA added. Cells were then placed on ice, and incubated for 30 minutes at 4°C in IM containing 50 µg/ml Alexa Fluor-555 labelled transferrin (Invitrogen). After washing with ice-cold PBS, prewarmed IM was added to the cells to allow internalization of transferrin, followed by incubation at 37°C for the times indicated. The reaction was stopped by putting the cells back on ice and washing with ice-cold PBS. Cells were then fixed and processed for confocal microscopy analysis as described for the immunofluorescence experiments.

### Flow cytometry

Internalization and recycling of transferrin were quantified by fluorescence-activated cell sorter (FACS), in HeLa cells transiently expressing GFP-Dmknδ5 or GFP alone. For these experiments, we used Alexa Fluor-647 labelled transferrin (Invitrogen) at the final concentration of 10 µg/ml in IM.

*Internalization assay* was performed as described above except that, after stopping the reaction, the non-internalized transferrin was removed by washing with ice-cold 0.2 M acetic acid (pH 2.8) containing 0.5 M NaCl. Cells were then washed with ice-cold PBS and detached with ice-cold PBS containing 5 mM EDTA. After washing with ice-cold PBS cells were resuspended in FACS buffer (2% BSA in PBS). Alexa Fluor-647 labelled transferrin uptake was measured by flow cytometry and the percentage of transferrin that was internalized at each time-point was calculated by subtracting background (fluorescence of cells subjected to acid wash without allowing internalization) and then normalized by the total amount of transferrin prebound at +4°C.

For *recycling experiments*, cells depleted of endogenous transferrin were incubated for 15 minutes at 37°C with prewarmed Alexa Fluor-647 labelled transferrin in IM. Cells were then placed on ice and washed with ice-cold PBS. Then, prewarmed IM was added followed by incubation at 37°C for the times indicated. Cells were then placed on ice, washed with ice-cold-PBS, and detached with trypsin. Harvested cells were washed in ice-cold-PBS and resuspended in FACS buffer. The amount of the fluorescent transferrin remained (non-released) in cells was measured by flow cytometry and expressed as the percentage of the initial intracellular transferrin amount detected in cells (100%, time 0 of recycling), in each experimental condition. Flow cytometry and data collection were performed on a FACSCalibur cell sorter (BD Biosciences). Data analysis was done using the WinMDI free software.

## Results

### The Dmknδ isoform family

**Figure 1:**
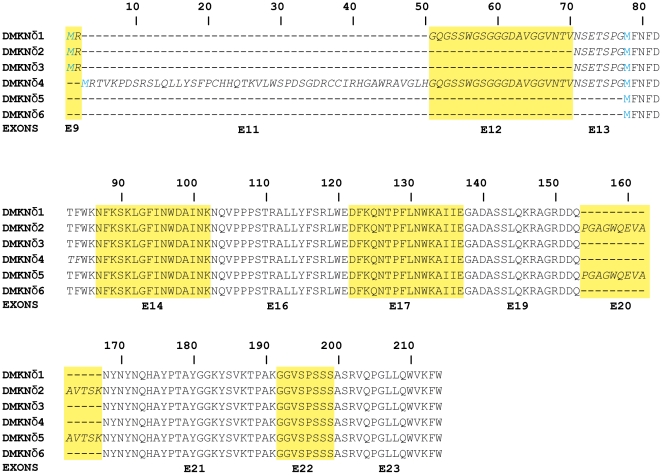
Organization of human Dmknδ isoform family. Amino-acid sequences encoded by different exons are individualized by yellow or white boxes, and the corresponding exon number is indicated at the bottom. The amino-acid sequences encoded by the alternative exons 9, 11, 12 and 20 are in italic. The putative first methionine that can be at amino-acid position 1, 3 or 76, is indicated in blue.

From the nine Dmknδ variants we previously cloned from human epidermis [3], we could deduce the sequence of 6 hypothetical proteins. All of them share a 123-amino-acid-length minimal sequence, and could be distinguished by 3 putative first methionine and additional sequences encoded by the alternative exons (Figure 1 and [3]). Unlike the other Dmkn groups of transcripts, Dmknδ mRNAs were shown to be ubiquitously expressed [3]. The following experiments presented in this paper which were carried out in order to characterize Dmknδ function, were performed with the Dmknδ5 isoform. The Dmknδ5 protein displays the minimal sequence present in all the Dmknδ plus the amino acids encoded by the alternative exon 20.

### Identification of Rab5 as a binding partner for Dmknδ5

**Figure 2:**
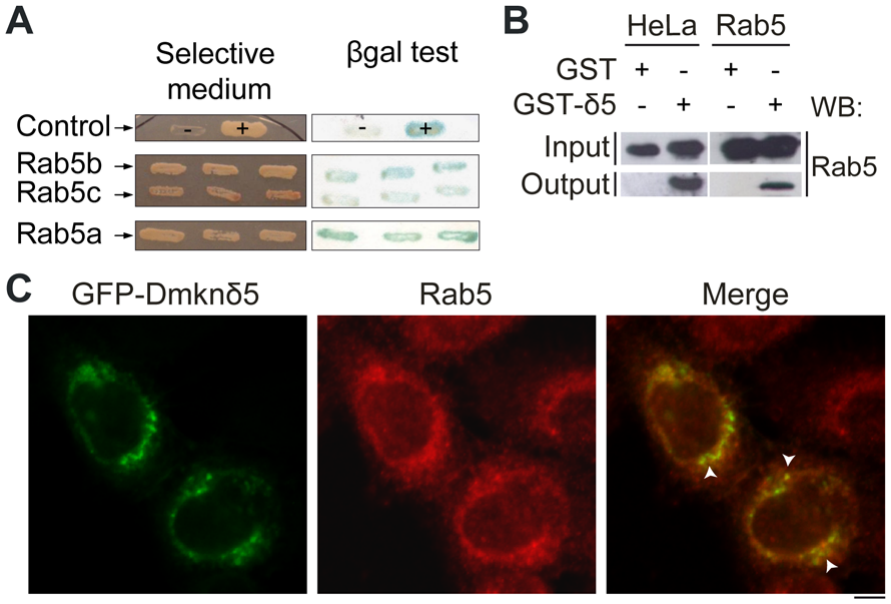
Rab5 is a partner of Dmknδ5. *A.* By yeast two-hybrid screening, positive clones were identified as Rab5b and Rab5c. Rab5a, subsequently tested, was also able to grow on selective medium and was positive for the β–galactosidase filter assay. Three representative clones of each double transformant corresponding to Dmknδ5/Rab5b, -c or -a are shown. *B.* GST-Dmknδ5 fusion protein or GST alone were captured on glutathione-sepharose beads before loading HeLa protein extract (HeLa) or purified recombinant wild-type Rab5 (Rab5). Proteins initially loaded onto the column (input) or eluted from the column (output) were detected by immunoblotting with an antibody directed against Rab5. *C.* HeLa cells were transiently transfected with GFP-Dmknδ5 (green) and processed for immunofluorescence analysis using an anti-Rab5 antibody (red). Representative transfected cells are shown, where GFP-Dmknδ5 is found in punctate structures (arrowheads) partially colocalized with endogenous Rab5.

The peptide sequences of the Dmknδ isoforms did not reveal any similarity with known functional domains. In order to gain an insight into Dmknδ function, we looked for potential partners using yeast two-hybrid analysis. We screened a human keratinocyte cDNA library with Dmknδ5 as a bait and obtained 5 clones growing on selective medium and positive for the β-galactosidase reporter gene assay. Four of them corresponded to full-length Rab5c and one to full-length Rab5b (Figure 2A). The small GTPase Rab5 having three isoforms that share 90% of sequence identity [16], we used the yeast two-hybrid system and found that the third isoform, Rab5a, was also able to interact with Dmknδ5 (Figure 2A). All further experiments were carried out using the Rab5b isoform. In order to confirm the interaction between Dmknδ5 and Rab5, we performed GST pull-down assays using bacterially expressed recombinant Dmknδ5. GST-Dmknδ5 was able to retain Rab5, either present in HeLa protein extract or produced as recombinant (Figure 2B). These data indicated that Dmknδ5 interacts with the endogenous Rab5 and confirmed that the interaction is direct. As we could not obtain Dmknδ specific antibody, we further analysed the localization of the endogenous Rab5 and the Dmknδ5 by confocal microscopy performed on HeLa cells expressing a GFP-tagged Dmknδ5. Rab5 was distributed throughout the cell body, with accumulation at the nuclear periphery (Figure 2C, *middle panel*) as previously described [22]. GFP-Dmknδ5 was detected as diffuse in the cytosol as well as concentrated in puncta localized in the perinuclear region (Figure 2C, *left panel*) where it partially colocalized with endogenous Rab5 (Figure 2C, *arrowheads*). The size of these structures, 0.5 to 1 µm, is typical of endosomal vesicles [19]. The expression of GFP alone only induced a diffuse cytosolic green labelling (data not shown), proving that the vesicle staining was related to Dmknδ5 expression.

### The domain of Dmknδ5 responsible for the interaction with Rab5 resides in the N-terminus region of the protein

**Figure 3:**
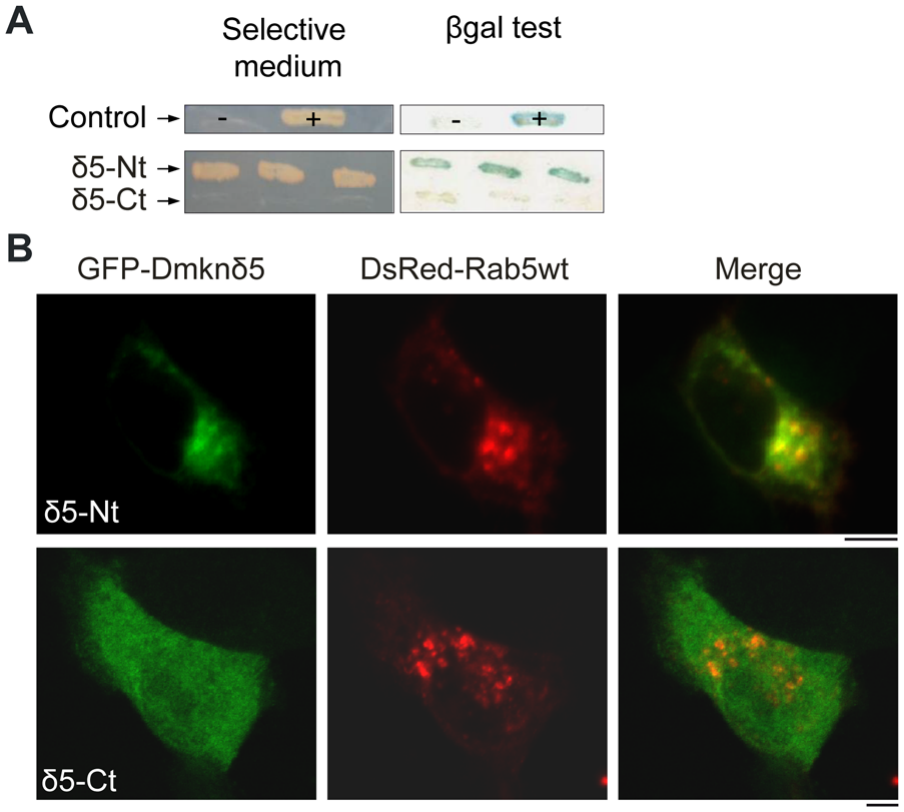
The N-terminal region of Dmknδ5 is responsible for the interaction with Rab5. *A.* δ5-Nt and δ5-Ct were tested for interaction with Rab5 using the yeast two-hybrid system. Expression of the reporter genes assay is shown for three representative clones of each double transformant (δ5-Nt/Rab5 and δ5-Ct/Rab5). *B.* HeLa cells were transiently transfected with DsRed-Rab5wt (red) and GFP-δ5-Nt or GFP-δ5-Ct (green) and observed by confocal microscopy. Bar, 5 µm

To specify the domain of Dmknδ5 involved in the interaction with Rab5, we constructed cDNAs encoding the N-terminus (Nt) or the C-terminus (Ct) of the protein, encompassing amino-acid residues 1 to 76 and 77 to 137, respectively. These constructs were assayed for interaction with Rab5, first by using the yeast two-hybrid system. As shown in figure 3A, only the clones expressing Dmknδ5-Nt grew on selective medium and expressed an active LacZ reporter gene. Thus, Dmknδ5 interacts with the small GTPase via its first 76 amino-acid residues. We then investigated the subcellular localization of both Dmknδ5 regions by confocal microscopy analysis of HeLa cells co-transfected with GFP-Dmknδ5-Nt or -Ct and DsRed-Rab5wt (Figure 3B). We found that GFP-Dmknδ5-Nt was localized in large endosome*-*like structures that were also positive for DsRed-Rab5wt, whereas GFP-Dmknδ5-Ct showed a diffuse cytosolic pattern of expression and never colocalized with the DsRed-Rab5wt-positive large endosomes. These results are consistent with the yeast two-hybrid assay and suggest that the vesicular location of Dmknδ5 is associated with the interaction of its N-terminus with Rab5.

### Dmknδ5 appears to be involved early in the endocytic pathway

**Figure 4:**
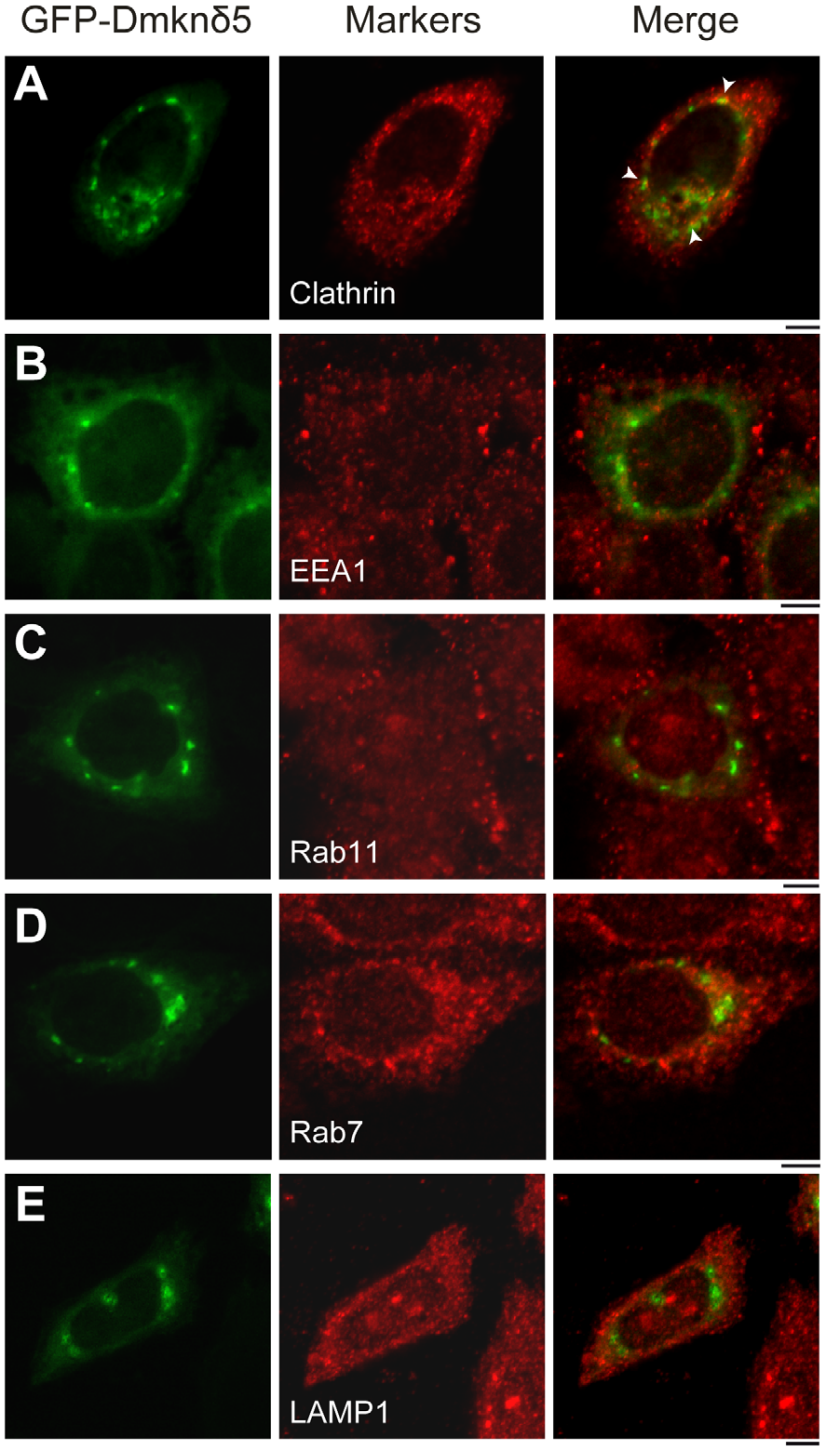
Characterization of Dmknδ5 positive vesicles. HeLa cells transiently transfected with GFP-Dmknδ5 (green) were processed for immunofluorescence analysis using antibodies directed against the endogenous organelle markers (red) clathrin (*A*), EEA1 (*B*), Rab11 (*C*), Rab7 (*D*) or LAMP1 (*E*). Cells were then visualized by confocal microscopy. Colocalization with GFP-Dmknδ5 was obvious only with clathrin as seen in the merged images (*A, arrowheads*). Bars, 5 µm

To further characterize the nature of GFP-Dmknδ5 positive structures, we analysed its co-localization in HeLa cells with several well-characterized organelle markers of the endocytic pathway (Figure 4). We first checked that transient expression of GFP-Dmknδ5 had no impact on the subcellular localization of these proteins. We next found that GFP-Dmknδ5 colocalized with clathrin on vesicles (Figure 4A, *arrowheads*). Moreover, GFP-Dmknδ5 never colocalized with the Rab5 effector EEA1, suggesting that the GFP-Dmknδ5 vesicles positive for Rab5 are distinct from early endosomes (Figure 4B). We also tested the late endosomal marker Rab7, the lysosomal protein LAMP1 and the recycling endosomal Rab11 and never noted any colocalization with GFP-Dmknδ5 (Figure 4C–E). Dmknδ5 could thus play its role in the endocytic pathway, as early as the formation of clathrin-coated vesicles.

**Figure 5:**
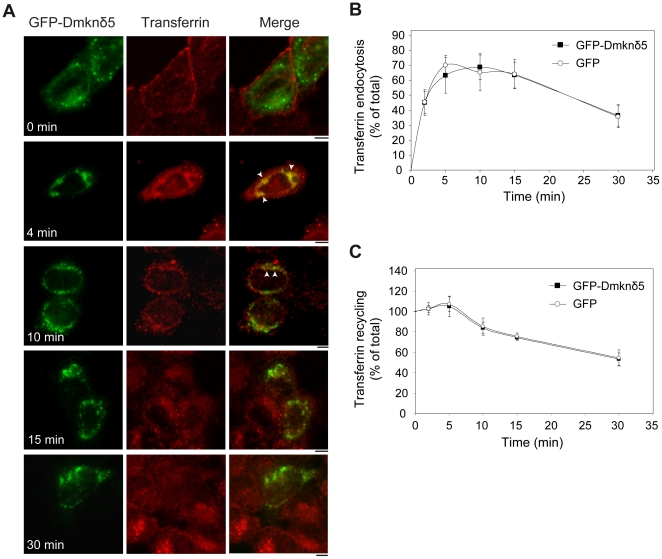
Dmknδ5 colocalizes early with endocytosed transferrin and does not influence transferrin uptake or recycling kinetics. *A*. HeLa cells transiently transfected with GFP-Dmknδ5 were incubated with AlexaFluor-555 conjugated-transferrin at 4°C (*0 min*). Transferrin uptake was then carried out for 4, 10, 15 or 30 min at 37°C, as indicated. Localization of GFP-Dmknδ5 (green) and Alexa Fluor-555 conjugated-transferrin (red) was then observed by confocal microscopy. Arrowheads show colocalization between GFP-Dmknδ5 and transferrin. Bars, 5 µm. *B, C.* Kinetics of endocytosis (*B*) and recycling (*C*) of transferrin in HeLa cells transiently expressing GFP-Dmknδ5 (black square) or GFP alone (open circle). For transferrin endocytosis, results are expressed as the percentage of internalized transferrin with respect to the prebound transferrin at +4°C (*B*). For recycling of intracellular transferrin, results are expressed as the percentage of initial (time 0, 100%) intracellular tranferrin (*C*). In *B* and *C*, the graphs are mean ± SD of three independent experiments.

In order to confirm these results, we performed pulse-chase experiments of Alexa-labelled transferrin in HeLa cells untransfected or transiently expressing the GFP-Dmknδ5 (Figure 5A). At the beginning of the chase (0 min), transferrin labelling was detected on the plasma membrane. Fluorescent transferrin was subsequently internalized and accumulated in big perinuclear puncta formed by the transient expression of GFP-Dmknδ5 in the cytoplasm (Figure 5A, *4* *min, arrowheads*). The staining of the membrane was no longer visible. After ten minutes of chase, colocalization of transferrin and GFP-Dmknδ5 strongly diminished (Figure 5A, *10 min, arrowheads*), and was no longer noticeable after 15 minutes of uptake. We next investigated whether the colocalization of GFP-Dmknδ5 with transferrin at early stages of endocytosis had an impact on the kinetic of transferrin uptake or recycling by fluorescence-activated cell sorter. No accelerated or delayed kinetics of internalization or recycling of transferrin was observed in HeLa cells expressing GFP-Dmknδ5 in comparison to HeLa cells expressing GFP alone (Figure 5B, C). Overall, these results confirm that Dmknδ5 plays its role upstream of early endosomes, probably during the clathrin-coated vesicle formation and/or transport to the sorting endosome, but does not modulate the kinetics of endocytosis or recycling of transferrin.

### Dmknδ5 interacts *in vitro* with both inactive and active forms of Rab5, but preferentially colocalizes with inactive Rab5 *in vivo*

**Figure 6:**
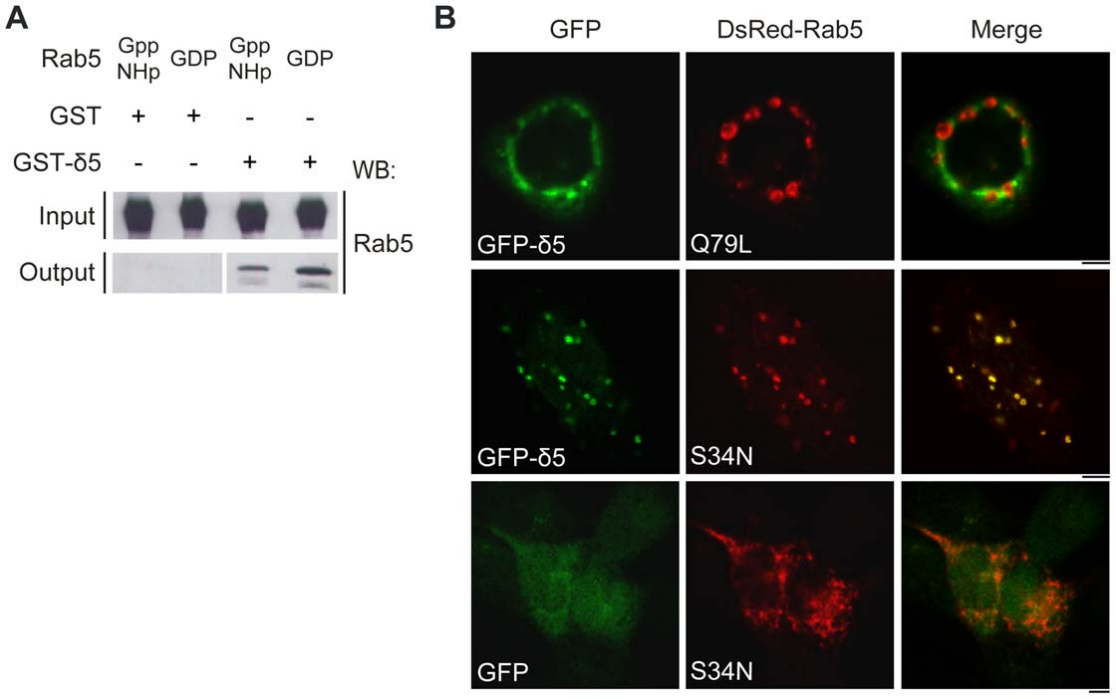
Dmknδ interacts with active and inactive Rab5. *A.* After capture of GST-Dmknδ5 fusion protein or GST alone on glutathione-sepharose beads, purified recombinant wild-type Rab5 (Rab5), incubated beforehand with GppNHp (active conformation) or GDP (inactive conformation), was loaded. Proteins initially loaded onto the column (input) or eluted from the column (output) were detected by immunoblotting with an antibody directed against Rab5. *B.* HeLa cells were transiently transfected with GFP-Dmknδ5 (green) and DsRed-Rab5Q79L or DsRed-Rab5S34N (red), respectively. Cells were then visualized by confocal microscopy. Bars, 5 µm

Protein interaction with Rab5 is modulated according to the nucleotide status of the small GTPase. In order to determine whether Dmknδ5 interacts preferentially with the active or the inactive form of Rab5, we performed GST pull-down assays. Recombinant Rab5 was subjected to an exchange reaction to load it with either GppNHp or GDP and test its interaction with GST-Dmknδ5 immobilized on glutathione-sepharose beads. Both GppNHp and GDP-bound Rab5 were retained by GST-Dmknδ5 (Figure 6A). We then analysed the in vivo colocalization of Dmknδ5 with the constitutively active or inactive forms of Rab5. For this purpose, HeLa cells were co-transfected with GFP-Dmknδ5 and DsRed-Rab5Q79L, or DsRed-Rab5S34N. Expression of the constitutively active DsRed-Rab5Q79L induced the formation of giant early endosomes as previously described [19]. These structures appeared negative for GFP-Dmknδ5 which localized to smaller vesicles (Figure 6B, *upper panel*). In contrast, GFP-Dmknδ5 colocalized with DsRed-Rab5S34N to a large extent (Figure 6B, *middle panel*). Interestingly, the forced expression of Dmknδ5 seemed to modify the morphology and the localization of the structures positive for the dominant negative Rab5 mutant. HeLa cells expressing GFP and DsRed-Rab5S34N formed typical tubulo-vesicular structures consistent with the inability of the mutant to promote membrane fusion [19] (Figure 6B, *lower panel*). In contrast, GFP-Dmknδ5 and DsRed-Rab5S34N colocalized into big endosome-like vesicles (1–1.5 µm) scattered around the cytoplasm. Hence, Dmknδ5 is able to interact with both the active and the inactive forms of Rab5 in vitro, but preferentially localizes with the inactive mutant of Rab5 in vivo.

### Dmknδ5 modifies the balance between inactive/active Rab5 in HeLa cells

**Figure 7:**
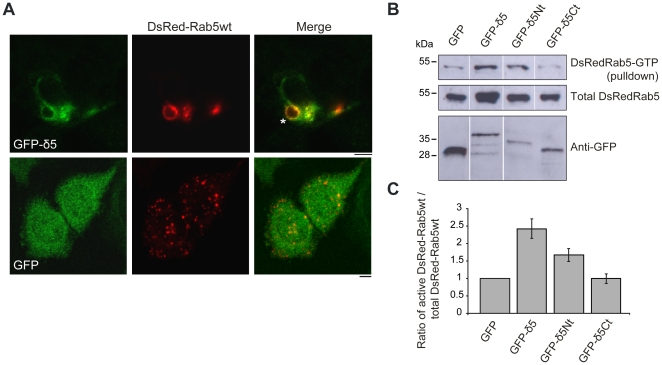
Dmknδ expression in HeLa cells modifies the balance Rab5-GTP/Rab5-GDP. *A*. HeLa cells transiently transfected with DsRed-Rab5wt (red) and GFP-Dmknδ5 or GFP alone (green), were observed by confocal microscopy. Bars, 5 µm *B.* DsRed-Rab5wt was co-expressed in HeLa cells with GFP, GFP-Dmknδ5 (GFP-δ5), GFP-Dmknδ5-Nt (GFP-δ5Nt), or GFP-Dmknδ5-Ct (GFP-δ5Ct) as indicated. *Top,* detection of DsRed-Rab5wt-GTP amount retained by R5BD-GST with the anti-Rab5 antibody. *Middle*, total amount of DsRed-Rab5wt protein present in each HeLa protein extract used for the pull-down experiment as determined by immunoblot with the anti-Rab5 antibody. *Bottom*, expression analysis of the different GFP-tagged constructs immunoblotted with the anti-GFP antibody. *C.* Quantification of active DsRed-Rab5wt-GTP. The ratio of DsRed-Rab5wt-GTP over total DsRed-Rab5wt was determined for each condition. Western blots from two independent experiments were analysed by densitometry. Values are mean ± s.e.m.

Interestingly, we saw that, when GFP-Dmknδ5 was expressed in addition to DsRed-Rab5wt, it induced the enlargement of the DsRed-Rab5wt positive structures (Figure 7A). The diameter of these vesicles increased from 1–1.5 µm when DsRed-Rab5wt was coexpressed with GFP alone (Figure 7A, *lower panel)*, to 2–3 µm when it was coexpressed with GFP-Dmknδ5 (Figure 7A *upper panel*). The large to giant vesicles induced by the transient expression of GFP-Dmknδ5 together with DsRed-Rab5wt are reminiscent of the giant endosomes caused by the constitutively active form Rab5Q79L [19]. This suggests that Dmknδ5 has an impact on the switch between the inactive (GDP-bound) and the active (GTP-bound) state of Rab5. To clarify this issue, we performed a GST pull-down assay based on the ability of the Rab5 binding domain (R5BD) of Rabaptin5, a Rab5 effector, to specifically link GTP-bound Rab5 [20], [23]. We produced GST-R5BD recombinant protein and used it to pull down Rab5-GTP in HeLa protein extract. HeLa cells were transfected with DsRed-Rab5wt and either GFP, GFP-Dmknδ5, GFP-Dmknδ5-Nt or GFP-Dmknδ5-Ct. We checked that all these GFP-tagged proteins were efficiently expressed in transfected HeLa cells, as shown in Figure 7B (*bottom panel*). Using an anti-Rab5 antibody, we confirmed the presence of the DsRed-Rab5wt in HeLa protein extracts (Figure 7B, *middle panel*). We also detected, with the same antibody, the DsRed-Rab5-GTP retained by the R5BD-GST beads (Figure 7B, *upper panel*). After quantification by densitometry, we found significantly increased levels of active DsRed-Rab5wt among cells expressing GFP-Dmknδ5 (∼2.5 fold) and, to a lesser extent, in cells expressing GFP-Dmknδ5-Nt (∼1.5 fold) (Figure 7C). This is consistent with our previous observation that GFP-Dmknδ5-Nt co-expressed with DsRed-Rab5wt did not induce the formation of giant vesicles (see Figure 3B). In contrast, the C-terminal domain of Dmknδ5 had no effect on DsRed-Rab5wt GTP level (Figure 7B, C). We can thus conclude that Dmknδ5 is able to modulate Rab5 activity by promoting its GTP loading. Moreover, the N-terminal region of Dmknδ5 does not seem to be fully functional, although it is able to interact with Rab5.

## Discussion

In this work, we describe for the first time the function of the ubiquitous Dmkn isoform, Dmknδ. By yeast two-hybrid and GST pull-down assays, we identified the small GTPase Rab5 as partner for Dmknδ5.

We observed that Dmknδ5 expression in HeLa cells modified some vesicle features. Dmknδ5 is sufficient to relocalize the Rab5S34N dominant negative mutant to large vesicular structures, scattered around the cytosol. The expression of Dmknδ5 could provoke endosome fusion in spite of the inhibitory effect of Rab5S34N. Such a rescue from Rab5S34N-mediated inhibition of endosome fusion has been described in the case of BHK cells transiently expressing the Rab5GEF Rabex5 [23]. Another feature of Dmknδ5 is its ability to enlarge the vesicles induced by Rab5wt expression in HeLa cells, from 1–1.5 µm to more than 2 µm. This vesicle size is reminiscent of that observed when the Rab5-GTP mutant is expressed in HeLa cells [19]. These data suggest that Dmknδ5 is able to promote early endosome fusion in vivo. Finally, we showed that Dmknδ5 activated Rab5 in vivo, by promoting GTP loading onto the small GTPase. This result is consistent with the observed Dmknδ5 impact on the morphology of Rab5 positive vesicles, and with Dmknδ5 preferential targeting of the GDP-bound Rab5 in vivo. Altogether, we found that Dmknδ5 activated Rab5 function and thus was involved in early endosome dynamics.

The vesicles induced by Dmknδ5 expression in HeLa cells colocalized only partially with Rab5. We thus investigated other known organelle markers in order to identify these Dmknδ5 structures. We found colocalization only with clathrin, which is present on plasma membrane and clathrin-coated vesicles. The Dmknδ5-positive vesicles never contained the early endosomal marker EEA1. These results were supported by the analysis of fluorescently labelled transferrin endocytosis. Transferrin was present in the same structures as Dmknδ5, from 0 to 10 minutes of chase, which corresponds to the transport of transferrin from the plasma membrane to the early endosome. Colocalization was reduced after 10 minutes of uptake, a time reported to match the association of Rab5 with EEA1 positive endosomes [24]. Finally, colocalization was no longer visible 15 minutes after the beginning of the chase, a time corresponding to transferrin transfer into structures positive for Rab5 and Rab11 [25]. Therefore, Dmknδ5 positive vesicles seem to transport transferrin from the plasma membrane to the sorting endosomes. However, we did not find any difference in the kinetics of the transferrin uptake or recycling between HeLa cells expressing GFP-Dmknδ5 or not.

We also investigated the specificity of Dmknδ5 binding towards Rab5 nucleotide state. In vitro, we found that it could bind to either the active GTP-bound or the inactive GDP-bound form of Rab5. Similar biochemical properties have been reported in the case of ALS2, Varp or the RIN family [26], [27], [28], [29], [30], all these proteins being identified as Rab5GEFs.

We identified the three Rab5 isoforms as partners for the Dmknδ5. Currently, few data comparing these three proteins are available. In a large scale analysis of Rab protein distribution, Gurkan et al. found that, despite their high homology, Rab5a, b and c exhibited distinct tissue distribution [17]. Moreover, although the three isoforms play their major role in the early endocytic pathway [16], they could be specifically regulated [31], [32] and differently involved in some processes [33], [34]. Their specificity of function may also reside in particular affinity with some partners, like GEF. It has recently been shown that Rin1 and Gapex-5, two Rab5 GEFs, could bind to either a single Rab or indifferently the three isoforms, respectively [15]. Dmknδ5, like Gapex-5, showed no significant specificity towards isoforms.

Overall, these in vivo and in vitro experimental results strongly suggest that Dmknδ5 acts like a GEF for Rab5. However, its amino-acid sequence lacks the well characterized VPS9 domain which is common to all Rab5 GEFs and required for the nucleotide exchange reaction [35]. Such properties have recently been described for another Rab5 partner, the caveolin-1 [36]. The authors hypothesized that caveolin-1 may recruit Rab5-GEF or promote its function onto Rab5, by direct binding. Dmknδ5 might have the same properties.

Our yeast two-hybrid screening did not allow us to identify a molecular partner for Dmknδ5 other than Rab5. However, this hypothesis is not excluded. Dmknδ5 binds to Rab5 via its N-terminal domain but coexpression of GFP-Dmknδ5-Nt and DsRed-Rab5wt in HeLa cells did not lead to formation of giant structures such as we observed when the full length GFP-Dmknδ5 was expressed instead of the truncated protein. Consistent results were found with the R5BD pull-down assay, showing that Rab5 activation by Dmknδ5 is reduced when only the N-terminal domain of the protein is expressed. Thus, the N-terminal part of Dmknδ5 is necessary but not sufficient for full function on Rab5 activation. Consequently, Dmknδ5 may interact, via its C-terminal domain, with another partner accounting for its modulation of Rab5 activation. Such an interaction may also be consistent with the vesicular location of Dmknδ5, which is not always correlated with the Rab5 one.

In conclusion, we found that Dmknδ5 is a new actor of the early endocytic pathway. Its direct interaction with Rab5 leads to activation of the small GTPase. We also showed that Dmknδ5 is involved in endocytosis of transferrin. Further studies will help to determine which molecular mechanisms and other potential partners are involved in this process.

## References

[1] Matsui T, Hayashi-Kisumi F, Kinoshita Y, Katahira S, Morita K (2004). Identification of novel keratinocyte-secreted peptides dermokine-alpha/-beta and a new stratified epithelium-secreted protein gene complex on human chromosome 19q13.1.. Genomics.

[2] Moffatt P, Salois P, St-Amant N, Gaumond MH, Lanctot C (2004). Identification of a conserved cluster of skin-specific genes encoding secreted proteins.. Gene.

[3] Toulza E, Galliano MF, Jonca N, Gallinaro H, Mechin MC (2006). The human dermokine gene: description of novel isoforms with different tissue-specific expression and subcellular location.. J Invest Dermatol.

[4] Bazzi H, Fantauzzo KA, Richardson GD, Jahoda CA, Christiano AM (2007). Transcriptional profiling of developing mouse epidermis reveals novel patterns of coordinated gene expression.. Dev Dyn.

[5] Naso MF, Liang B, Huang CC, Song XY, Shahied-Arruda L (2007). Dermokine: an extensively differentially spliced gene expressed in epithelial cells.. J Invest Dermatol.

[6] Brandenberger R, Wei H, Zhang S, Lei S, Murage J (2004). Transcriptome characterization elucidates signaling networks that control human ES cell growth and differentiation.. Nat Biotechnol.

[7] Kimura K, Wakamatsu A, Suzuki Y, Ota T, Nishikawa T (2006). Diversification of transcriptional modulation: large-scale identification and characterization of putative alternative promoters of human genes.. Genome Res.

[8] Wakamatsu A, Kimura K, Yamamoto J, Nishikawa T, Nomura N (2009). Identification and Functional Analyses of 11 769 Full-length Human cDNAs Focused on Alternative Splicing.. DNA Res.

[9] Zerial M, McBride H (2001). Rab proteins as membrane organizers.. Nat Rev Mol Cell Biol.

[10] Stenmark H (2009). Rab GTPases as coordinators of vesicle traffic.. Nat Rev Mol Cell Biol.

[11] Pfeffer SR (2005). Structural clues to Rab GTPase functional diversity.. J Biol Chem.

[12] Schwartz SL, Cao C, Pylypenko O, Rak A, Wandinger-Ness A (2007). Rab GTPases at a glance.. J Cell Sci.

[13] Nielsen E, Severin F, Backer JM, Hyman AA, Zerial M (1999). Rab5 regulates motility of early endosomes on microtubules.. Nat Cell Biol.

[14] Lanzetti L, Palamidessi A, Areces L, Scita G, Di Fiore PP (2004). Rab5 is a signalling GTPase involved in actin remodelling by receptor tyrosine kinases.. Nature.

[15] Chen PI, Kong C, Su X, Stahl PD (2009). Rab5 isoforms differentially regulate the trafficking and degradation of Epidermal Growth Factor Receptors.. J Biol Chem.

[16] Bucci C, Lutcke A, Steele-Mortimer O, Olkkonen VM, Dupree P (1995). Co-operative regulation of endocytosis by three Rab5 isoforms.. FEBS Lett.

[17] Gurkan C, Lapp H, Alory C, Su AI, Hogenesch JB (2005). Large-scale profiling of Rab GTPase trafficking networks: the membrome.. Mol Biol Cell.

[18] Christoforidis S, Zerial M (2000). Purification and identification of novel Rab effectors using affinity chromatography.. Methods.

[19] Stenmark H, Parton RG, Steele-Mortimer O, Lutcke A, Gruenberg J (1994). Inhibition of rab5 GTPase activity stimulates membrane fusion in endocytosis.. Embo J.

[20] Stenmark H, Vitale G, Ullrich O, Zerial M (1995). Rabaptin-5 is a direct effector of the small GTPase Rab5 in endocytic membrane fusion.. Cell.

[21] Torres VA, Mielgo A, Barila D, Anderson DH, Stupack D (2008). Caspase 8 promotes peripheral localization and activation of Rab5.. J Biol Chem.

[22] Chavrier P, Parton RG, Hauri HP, Simons K, Zerial M (1990). Localization of low molecular weight GTP binding proteins to exocytic and endocytic compartments.. Cell.

[23] Zhu H, Zhu G, Liu J, Liang Z, Zhang XC (2007). Rabaptin-5-independent membrane targeting and Rab5 activation by Rabex-5 in the cell.. Mol Biol Cell.

[24] Yoon HY, Lee JS, Randazzo PA (2008). ARAP1 regulates endocytosis of EGFR.. Traffic.

[25] Sonnichsen B, De Renzis S, Nielsen E, Rietdorf J, Zerial M (2000). Distinct membrane domains on endosomes in the recycling pathway visualized by multicolor imaging of Rab4, Rab5, and Rab11.. J Cell Biol.

[26] Kajiho H, Saito K, Tsujita K, Kontani K, Araki Y (2003). RIN3: a novel Rab5 GEF interacting with amphiphysin II involved in the early endocytic pathway.. J Cell Sci.

[27] Otomo A, Hadano S, Okada T, Mizumura H, Kunita R (2003). ALS2, a novel guanine nucleotide exchange factor for the small GTPase Rab5, is implicated in endosomal dynamics.. Hum Mol Genet.

[28] Saito K, Murai J, Kajiho H, Kontani K, Kurosu H (2002). A novel binding protein composed of homophilic tetramer exhibits unique properties for the small GTPase Rab5.. J Biol Chem.

[29] Tall GG, Barbieri MA, Stahl PD, Horazdovsky BF (2001). Ras-activated endocytosis is mediated by the Rab5 guanine nucleotide exchange activity of RIN1.. Dev Cell.

[30] Zhang X, He X, Fu XY, Chang Z (2006). Varp is a Rab21 guanine nucleotide exchange factor and regulates endosome dynamics.. J Cell Sci.

[31] Chiariello M, Bruni CB, Bucci C (1999). The small GTPases Rab5a, Rab5b and Rab5c are differentially phosphorylated in vitro.. FEBS Lett.

[32] Callaghan J, Nixon S, Bucci C, Toh BH, Stenmark H (1999). Direct interaction of EEA1 with Rab5b.. Eur J Biochem.

[33] Wainszelbaum MJ, Proctor BM, Pontow SE, Stahl PD, Barbieri MA (2006). IL4/PGE2 induction of an enlarged early endosomal compartment in mouse macrophages is Rab5-dependent.. Exp Cell Res.

[34] Barbieri MA, Roberts RL, Gumusboga A, Highfield H, Alvarez-Dominguez C (2000). Epidermal growth factor and membrane trafficking. EGF receptor activation of endocytosis requires Rab5a.. J Cell Biol.

[35] Delprato A, Lambright DG (2007). Structural basis for Rab GTPase activation by VPS9 domain exchange factors.. Nat Struct Mol Biol.

[36] Hagiwara M, Shirai Y, Nomura R, Sasaki M, Kobayashi K (2009). Caveolin-1 activates Rab5 and enhances endocytosis through direct interaction.. Biochem Biophys Res Commun.

